# Evaluating Baculovirus as a Vector for Human Prostate Cancer Gene Therapy

**DOI:** 10.1371/journal.pone.0065557

**Published:** 2013-06-06

**Authors:** Stephanie L. Swift, Guillermo C. Rivera, Vincent Dussupt, Regina M. Leadley, Lucy C. Hudson, Corrina MA de Ridder, Robert Kraaij, Julie E. Burns, Norman J. Maitland, Lindsay J. Georgopoulos

**Affiliations:** 1 Yorkshire Cancer Research Unit, Department of Biology, University of York, Heslington, York, United Kingdom; 2 Department of Urology, Erasmus MC, Rotterdam, The Netherlands; IPO, Inst Port Oncology, Portugal

## Abstract

Gene therapy represents an attractive strategy for the non-invasive treatment of prostate cancer, where current clinical interventions show limited efficacy. Here, we evaluate the use of the insect virus, baculovirus (BV), as a novel vector for human prostate cancer gene therapy. Since prostate tumours represent a heterogeneous environment, a therapeutic approach that achieves long-term regression must be capable of targeting multiple transformed cell populations. Furthermore, discrimination in the targeting of malignant compared to non-malignant cells would have value in minimising side effects. We employed a number of prostate cancer models to analyse the potential for BV to achieve these goals. *In vitro*, both traditional prostate cell lines as well as primary epithelial or stromal cells derived from patient prostate biopsies, in two- or three-dimensional cultures, were used. We also evaluated BV *in vivo* in murine prostate cancer xenograft models. BV was capable of preferentially transducing invasive malignant prostate cancer cell lines compared to early stage cancers and non-malignant samples, a restriction that was not a function of nuclear import. Of more clinical relevance, primary patient-derived prostate cancer cells were also efficiently transduced by BV, with robust rates observed in epithelial cells of basal phenotype, which expressed BV-encoded transgenes faster than epithelial cells of a more differentiated, luminal phenotype. Maximum transduction capacity was observed in stromal cells. BV was able to penetrate through three-dimensional structures, including *in vitro* spheroids and *in vivo* orthotopic xenografts. BV vectors containing a nitroreductase transgene in a gene-directed enzyme pro-drug therapy approach were capable of efficiently killing malignant prostate targets following administration of the pro-drug, CB1954. Thus, BV is capable of transducing a large proportion of prostate cell types within a heterogeneous 3-D prostate tumour, can facilitate cell death using a pro-drug approach, and shows promise as a vector for the treatment of prostate cancer.

## Introduction

In the United Kingdom, over 40,000 new cases of prostate cancer are diagnosed every year and 11,000 patients die as a direct result of this disease (Source: Office for National Statistics). The human prostate gland is a heterogeneous environment, primarily composed of epithelial and stromal cell types. The epithelial compartment consists of a bilayer of cells: a continuous basal layer of undifferentiated cells that are in contact with the basement membrane, overlaid with differentiated secretory luminal cells [1]. The basal epithelial layer represents the most proliferative compartment [2]. While the bulk of most prostate tumours comprises cells of epithelial origin [3], multiple cell types have the capacity to contribute towards malignancy, and must be eradicated in order to achieve an effective clinical outcome.

Typically, the stage of prostate disease and the responsiveness of the tumour to androgens are two key factors that direct treatment strategies. While localised prostate tumours can be surgically resected via radical prostatectomy, this is often accompanied by undesirable side effects. An alternative approach, androgen ablation therapy, removes the supply of androgens that many prostate tumours rely on for growth and survival; however, the recurrence of more aggressive castration-resistant prostate cancer (CRPC) is a common outcome. Since CRPC is ultimately resistant to radiotherapy and chemotherapy, novel strategies are essential to provide improved outcomes for prostate cancer patients. Gene therapy is one potential solution, and would ideally be positioned to target both primary and secondary prostate tumours following systemic administration.

While many different vectors have been evaluated for safety and efficacy in gene therapy trials [4], the use of non-human, non-pathogenic viruses, such as the baculovirus, *Autographa californica* Multiple NucleoPolyHedrovirus (*Ac*MNPV), is a relatively understudied area. BV is a double-stranded DNA virus that naturally infects insect hosts, and although BV can efficiently transduce mammalian cells, productive replication is not possible due to the requirement for insect-specific cellular machinery [5], [6]. As a gene therapy vector, BV has several inherent advantages. The bulk of the human population has no previous exposure to BV and is thus immunologically naive. The BV capsid is capable of expanding to accommodate large transgene inserts, and the natural tropism of baculovirus is easily modified. BV is able to transduce both actively-dividing and quiescent mammalian cells, a feature that has particular relevance in the treatment of prostate cancer, a slow-growing tumour [7]. The presence of baculovirus, even at a high multiplicity of infection (MOI), is not toxic and does not affect cell growth and differentiation potential [8], [9]. Finally, extensive long-term safety studies, undertaken in the 1960’s when baculovirus emerged as an important prospect as a biological insecticide, have proven baculovirus harmless to man [10].

The ability of BV to deliver functionally sustainable transgene expression in mammalian cells was first demonstrated in 1995 [11]. Since then, *Ac*MNPV has proven capable of transducing a wide range of mammalian cell types, including human primary neurons [12], liver [11], mesenchymal stem cells [13] and embryonic stem cells [9]. While the earliest attempts to achieve BV-mediated gene transfer *in vivo* failed due to vector inactivation by serum components, such as complement [14], efficient baculovirus transduction can be achieved in the presence of chemical- or protein-based complement inhibitors, either as co-inocula [15], [16], [17] or incorporations into the BV envelope protein [17], [18], [19]. BV has been successfully used as a preclinical gene therapy vector *in vivo* [12], [20], [21], [22], [23], and in the context of cancer has successfully treated murine gastric xenografts [24].

Here, we evaluate BV as a gene therapy vector for the treatment of human prostate cancer. We describe the use of BV to preferentially transduce and deliver both reporter and cell-lethal genes to malignant prostate samples of cell line and patient origin, in addition to three-dimensional cultures and *in vivo* murine tumours, with high efficiency.

## Materials and Methods

### Insect Cell Line Maintenance

Sf21 and Sf9 (Invitrogen) cell lines (isolated from the ovarian tissue of the fall army worm, *Spodoptera frugiperda*) were maintained in spinner hanging stir bar flasks at 27°C in Grace’s medium supplemented with 10% foetal calf serum (PAA Laboratories) and 0.1% (v/v) Pluronic® F-68 (Invitrogen). Monolayer cultures were maintained in the absence of Pluronic.

### Human Prostate Cell Line Maintenance

LNCaP (androgen-sensitive, well-differentiated prostate cancer cells derived from a lymph node metastasis) and PC-3 (androgen-independent, poorly-differentiated prostate cancer cells derived from a bone metastasis) were purchased from the American Type Culture Collection (ATCC, USA). PC346C (androgen-sensitive, well-differentiated prostate cancer cells derived from a primary prostate tumour) were established in the Erasmus Medical Centre, The Netherlands [25]. P4E6 (epithelial cells derived from a well-differentiated primary prostate tumour immortalised by transfection with HPV E6), PNT1A and PNT2C2 (non-malignant prostate cells from different patients, respectively, immortalised by transfection with SV40) were established in our laboratory [26], [27]. All cells were handled under good laboratory practice conditions in defined passage windows, were monthly certified free of mycoplasma and were genotyped (using the ATCC-approved Powerplex 1.2 system, Promega) to ensure authenticity. Cells were routinely passaged under previously described conditions [28].

### Primary Cell Culture

Patient prostate tissue was taken with informed and written consent and approval from the York Research Ethics Committee from patients undergoing transurethral resection of the prostate, cystectomy or radical prostatectomy. Epithelial cells were isolated as described previously [29] and selected for α_2_β_1_^hi^ expression using rapid adherence to type I Collagen-coated plates (BD Biosciences). Cells were co-cultured on Collagen I plasticware with irradiated STO murine feeder cells until growth was well established, in KSFM supplemented with 5 ng/ml EGF, 50 µg/ml bovine pituitary extract and 2 mM L-Glutamine (KSFMsup). To induce differentiation, cells were cultured in a 50∶50 (v/v) mixture of DMEM and Ham’s F12, supplemented with 10% FCS, 10 nM DHT and 2 mM L-Glutamine (DH10) for 4–6 days. Isolated stromal cells were propagated in T25 flasks in RPMI supplemented with 10% FCS and 2 mM L-Glutamine.

### Baculovirus Preparation

Recombinant baculovirus vectors were constructed using the BacVector 1000 kit (Novagen), according to the manufacturer’s instructions, with the custom-made pBAC64:CMV-EGFPCAT, pBAC64:CMV-EGFP or pBAC64:CMV-NTR-EGFP transfer plasmids. These plasmids have the backbone of pBAC4X-1 (Novagen) with insertions of the *polh* promoter and *gp64* gene coding sequences from pBACsurf-1 (Novagen) and the cytomegalovirus (CMV) immediate early promoter driving transcription of EGFP, an EGFPCAT fusion protein (BD Biosciences Clontech) or mNitroreductase(NTR)-IRES-EGFP expression cassettes. Recombinant viruses were subjected to three rounds of plaque purification and high titre stocks were grown by infecting Sf21 insect cells at MOI = 0.1 plaque forming units (PFU) per cell and harvesting virus supernatant after 5 days. Virus-containing cell supernatant was concentrated by ultracentrifugation at 24,000 RPM for 90 minutes at 4°C using a Beckman SW28 rotor and further purified by ultracentrifugation through a sucrose gradient at 24,000 RPM in a Beckman SW41 rotor. Virus particles were washed and resuspended in PBS (approximately 1/500 starting volume) and titrated using plaque assay on Sf9 cells. The particle:PFU ratio was generally within the range of 100–300 [30], [31], [32]. For transduction of prostate cells, virus was diluted in serum-free medium appropriate for each cell type, unless otherwise stated.

### Confocal Microscopy Analysis of Monolayers and Bilayers

For both monolayer and bilayer confocal experiments, cells were seeded onto 8-well chamber slides with a coverslip base (Lab-Tek) at an initial density of 2–5×10^4^ cells per well (pre-coated with 0.1 mg/ml poly-L-lysine for LNCaP cells). To generate a bilayer, medium was changed every 2 days until a dual layer of epithelial growth was achieved. BV-[CMV-EGFPCAT] or BV-[CMV-EGFP] particles were added at 500 PFU/cell for a specified length of time after which cells were washed to remove unbound virus and fixed immediately in 4% (w/v) paraformaldehyde. Cells were permeabilised in 0.5% (v/v) Triton-X 100 (Sigma) or 70% EtOH, then blocked in 10% serum. The BV capsid protein, vp39, was detected using a primary monoclonal antibody (P10C6), courtesy of Dr. L.E. Volkman, University of California, Berkeley, USA [33] and a goat anti-mouse Alexa Fluor 568 secondary antibody (Invitrogen). In bilayer experiments, antibody dilutions were prepared in 0.1% (w/v) Fraction V BSA (Sigma) to aid penetration in basal layers, while in monolayer experiments, antibody dilutions were prepared in PBS. For analysis of primary cell differentiation, HMWCK (Dako) antibody was added with the subsequent application of a secondary goat anti-mouse Alexa 568 antibody (Invitrogen). A second primary antibody (CK18-FITC (Sigma)) was then applied. Vectashield containing DAPI (Vector Lab) was added before visualising cells under fluorescence using a Zeiss LSM 510 meta confocal microscope.

### Flow Cytometry

Cells were plated at a density of 0.2–1×10^5^ per well of a 48-well plate and incubated at 37°C overnight. Cells were washed once in PBS before addition of virus diluted in serum-free medium appropriate for each cell type. After the desired length of time, virus was aspirated, cells washed once in serum-free medium and overlaid in serum-containing growth medium. Cells were detached using Trypsin:EDTA and resuspended in 0.02% (w/v) EDTA, with a minimum of 10,000 singlet events counted using a CyAn™ ADP flow cytometer.

### PC346C Spheroid Transduction

Spontaneously formed spheroids were harvested from the surface of a T25 flask containing PC346C cells and incubated for 10 minutes at 37°C in complete culture medium containing BV-[CMV-EGFP] at a concentration of 8.6×10^9^ PFU/ml. The spheroids were diluted in 2.5 ml of medium and re-plated into a T12.5 flask. Fluorescent images were captured using a Nikon Eclipse TE300 microscope at three days post-infection.

### Orthotopic Xenografts

Tumours were established by injection of 1×10^6^ PC346C cells orthotopically into the dorsolateral prostate of athymic nude mice (NMRI nu/nu, Taconic M&B A/S, Ry, Denmark) as described previously [34], in compliance with ethical procedures and under approval from the Erasmus Experimental Animal Center ethics committee. Tumour growth was monitored by transrectal ultrasonography using an adapted intravascular ultrasound system [35]. At tumour sizes between 80 and 120 mg, two doses (3×10^7^ or 1×10^8^ pfu) of BV-[CMV-EGFP] were administered intratumourally under anaesthesia. Animals were sacrificed 3 days later, tumours were harvested, fixed for 2 h in 2% (w/v) paraformaldehyde in PBS, sliced on a Vibratome (Campden Instruments, Loughborough, UK) into 70 µm thick sections and mounted on glass slides in Vectashield (Vector Lab). EGFP expression was visualised by confocal microscopy (Carl Zeiss, Benelux).

### Evaluation of Cell Viability by MTS Assay

Cells were plated at a density of 1×10^4^ cells per well in a 96 well plate and allowed to attach overnight. BV-[CMV-NTR-EGFP] was added to triplicate wells at MOI = 500 for 4 hours, washed and incubated for a further 20 hours before addition of CB1954 (20 µM for all primary samples and cell lines except for PC-3, for which 40 µM was used). After a further 48 h for cell lines or 30 h for primary cells, cell viability was measured using the CellTiter 96® Aqueous Assay Reagent (Promega) according to the manufacturer’s instructions and plates read using a BMG Labtech POLARstar OPTIMA plate reader. The proportion of dead cells was calculated by comparing to viable cells in an untransduced well. A duplicate set of wells were used for flow cytometry as described above to determine the frequency of positively transduced cells.

## Results

### BV Transduction is Most Efficient in Malignant Prostate Cell Lines Compared to Non-malignant Controls

**Figure 1:**
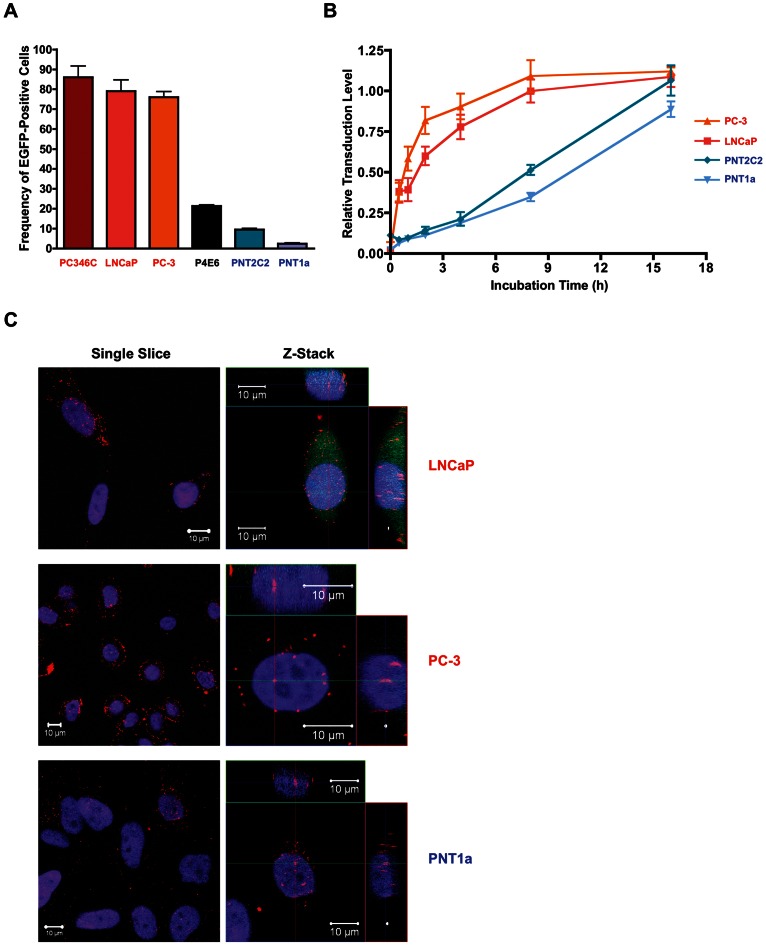
BV transduction of prostate cell lines. (**A**) Percentage of EGFP-positive cells following transduction of a panel of high grade malignant (red), low grade malignant (black) or non-malignant (blue) prostate cell lines with BV-[CMV-EGFPCAT] at MOI = 500 for 48 h. Error bars depict −/+ one standard deviation. (**B**) Relative expression levels of EGFP following transduction with BV-[CMV-EGFPCAT] at a saturating MOI (500 for LNCaP, PC3 and PNT1A, 1000 for PNT2C2). The percentage of EGFP-positive cells was normalised to the levels achieved following 48 h incubation in the presence of virus for each cell type (set to 1). Malignant cell lines: PC3 (▴), LNCaP (▪); Non-Malignant Cell Lines: PNT2C2 (♦), PNT1A (▾). Error bars depict −/+ one standard deviation. (**C**) Confocal microscopy images (single slice or Z-stack) of BV-transduced LNCaP, PC3 or PNT1A cells at 8 h post-transduction (MOI = 500). Red fluorescence indicates BV capsid (anti-vp39), nuclear staining in blue (DAPI) and BV-driven EGFP expression in green.

In order to determine the capacity of BV to differentially transduce malignant vs. non-malignant prostate targets, we evaluated the susceptibility of a panel of established human prostate cell lines to BV transduction. Cells were incubated with a recombinant BV vector modified to carry an EGFPCAT transgene (BV-[CMV-EGFPCAT]) using a fixed MOI of 500 and an incubation time of 48 h. Malignant cell lines with high metastatic potential, including LNCaP, PC3 and PC346C, were highly transduced (∼80% EGFP-positive cells), while non-malignant cell lines PNT1A and PNT2C2 were least transduced (up to 10% EGFP-positive cells) (Figure 1A). P4E6, a cell line derived from an initiated but early stage tumour, was in the intermediate range, with approximately 20% EGFP-positive cells (Figure 1A). Thus, the frequency of cells positively transduced by BV correlated with malignancy and metastatic potential.

To investigate the optimal transduction conditions for highly susceptible (LNCaP, PC-3) versus less susceptible (PNT1A and PNT2C2) prostate cell lines, we analysed both the MOI required to achieve transduction saturation and the kinetics of transduction at this MOI. Cells were incubated for 1 h in the presence of increasing doses of BV-[CMV-EGFPCAT], and the proportion of EGFP-positive cells was analysed after 24 h. The MOI required to achieve the maximum frequency of transduced cells was 500 PFU per cell for LNCaP, PC3 and PNT1A, and 1000 PFU per cell for PNT2C2 (data not shown). Using these saturating MOIs, the kinetics of transduction were investigated over time. Malignant prostate cell lines (LNCaP and PC-3) demonstrated rapid and efficient uptake of BV-[CMV-EGFPCAT], requiring only short incubation times (∼45 or ∼90 min, respectively) to reach 50% of the levels recorded at 48 h (Figure 1B). Conversely, non-malignant prostate cell lines (PNT1A and PNT2C2) required extended incubation times (∼10 h or ∼8 h, respectively) to achieve 50% of the levels at 48 h (Figure 1B).

One interpretation of these differences in transduction is that nuclear import of the BV capsid is defective in some cell types (reviewed by [36]). To evaluate this hypothesis in our prostate cell line models, the subcellular localisation of BV capsids was investigated using confocal microscopy at 8 h post-transduction in the highly susceptible malignant LNCaP and PC-3 cell lines and the less susceptible non-malignant PNT1A cell line. BV capsids were observed in the nucleus of all three cell types (Figure 1C), and visual analysis of multiple fields of view did not reveal any differences in the levels of nuclear capsid localisation. However, PC-3 cells had a higher level of surface-bound BV, whereas PNT1A cells had a higher level of BV in the cytoplasm (Figure 1C).

### Cells of Basal (Undifferentiated) Phenotype Are Transduced More Readily than Luminal (Differentiated) Cells in Primary Prostate Epithelial Cultures

**Figure 2:**
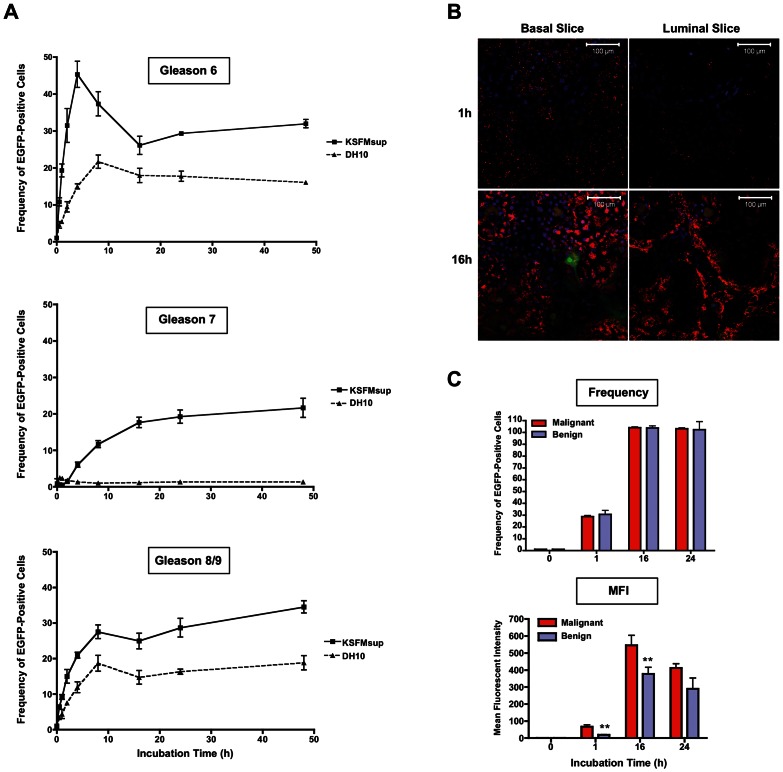
BV transduction of primary prostate epithelial cells. (**A**) Percentage of EGFP-positive cells at 48 h post-transduction with Bv-[CMV-EGFPCAT] at MOI = 500 for increasing lengths of time in three primary malignant prostate epithelial samples (Gleason 6, 7 or 8/9), maintained in a basal state by culture in KSFMsup (▪), or cultured in differentiating conditions in DH10 (▴). Error bars depict −/+ one standard deviation. (**B**) Localisation of BV capsids in primary epithelial cells from a Gleason 8/9 tumour grown in a bilayer, either 1 h or 16 h after incubation with Bv-[CMV-EGFPCAT] at MOI = 250. Red fluorescence indicates BV capsids detected with anti-vp39, nuclear DAPI staining is shown in blue and BV-driven EGFP expression in green. Confocal images of the upper layer of cells (luminal-like; at an average z-distance of 14.58 µm from the ventral position) and lower layer (basal-like; at an average z-distance of 6.47 µm from the ventral position) are shown. (**C**) Frequency (normalised to mock = 1%) or mean fluorescence intensity of EGFP-positive primary stromal cells derived from malignant or benign biopsies 24 h following transduction with Bv-[CMV-EGFPCAT] at MOI = 500.

In order to extend our evaluation of BV into a more biologically relevant situation, we employed primary patient biopsy-derived prostate epithelial cell cultures. Since the bulk of human prostate tumours comprises cells of epithelial origin, with both undifferentiated (basal) and well-differentiated (luminal) phenotypes present, we determined whether BV was capable of targeting both epithelial cell populations at different stages of differentiation. Primary prostate epithelial cells derived from three patient tumour biopsies (Gleason scores 6, 7 and 8/9) were cultured under two different conditions: in KSFMsup medium to maintain cells in a basal state, or in DH10 medium to induce differentiation, based on work by [37]. Changes in differentiation status under these distinct culture conditions were confirmed by immunofluorescent analysis of classical differentiation markers (basal markers: cytokeratins (CK) 1, 5, 10 and 14; luminal marker: CK18), based on work by [38] (Supplementary Figure 1). On a per-patient basis, cells cultured in KSFMsup or DH10 were incubated with BV-[CMV-EGFPCAT] for increasing lengths of time and analysed by flow cytometry at 48 h. Epithelial differentiation reduced transduction frequencies overall when the cells were incubated with virus for less than 10 h: for example, in cells derived from a Gleason 6 tumour, the maximum level of infection achieved in basal (KSFMsup) cells at 4 h (45.33±3.55%) was considerably higher than the peak of infection in luminal (DH10) cells (21.69±1.78%) at 8 h (Figure 2A). Over longer periods of BV incubation, the difference in transduction levels between KSFMsup- and DH10-cultured cells became less pronounced, but remained lower in differentiated (DH10) cell populations (Figure 2A).

In order to further investigate the rapid transduction of basal epithelial cells with BV, we employed an *in vitro* cell bilayer model, which mimicks the cellular structure of the prostate gland. The bilayer comprised a confluent layer of basal epithelial cells (CK1/5/10/14+) overlaid with an imperfect upper layer of cells with a more differentiated, luminal phenotype (CK18+) [28], [38]. Primary malignant prostate epithelial cells derived from a Gleason score 7 tumour were allowed to form a bilayer, then incubated with BV-[CMV-EGFPCAT] for 1 h or 16 h and visualised using confocal microscopy. After incubation with BV for 1 h, virus capsids were visualised in the basal layer in a punctate staining pattern, but were not observed in the luminal layer (Figure 2B). Following a longer period of incubation (16 h), virus capsids were detected in both basal and luminal layers: however, notable areas in the luminal layer remained devoid of virus capsids (Figure 2B).

### BV can Rapidly and Efficiently Transduce Primary Prostate Stromal Cells

Epithelial and stromal populations represent the two major cellular components of prostate tumours. While epithelial cells typically comprise the bulk of the malignant cell population, stromal cells have been implicated in a reciprocal feedback loop that supports the growth and maintenance of tumour cells [39], [40]. This suggests that a successful prostate cancer therapy should include targeted eradication of the malignancy-associated stromal compartment. We therefore investigated the ability of BV to transduce cells of stromal origin. Stromal cells derived from malignant or non-malignant primary prostate biopsies were incubated with BV-[CMV-EGFPCAT] for 1, 16 or 24 hours and analysed by flow cytometry. After 1 h, approximately 30% of cells were transduced by BV, while after 16 h incubation, every stromal cell was positively transduced by BV (Figure 2C). No difference was observed in the frequency of positively-transduced stromal cells based on malignancy; however, on a per-cell basis, stromal cells derived from malignant prostate biopsy samples exhibited a greater mean fluorescence intensity than those derived from benign biopsy samples at early timepoints (Figure 2C). Stromal cells were therefore highly susceptible to BV transduction.

### BV can Successfully Transduce 3-D Prostate Spheroids and Orthotopic Xenografts

**Figure 3:**
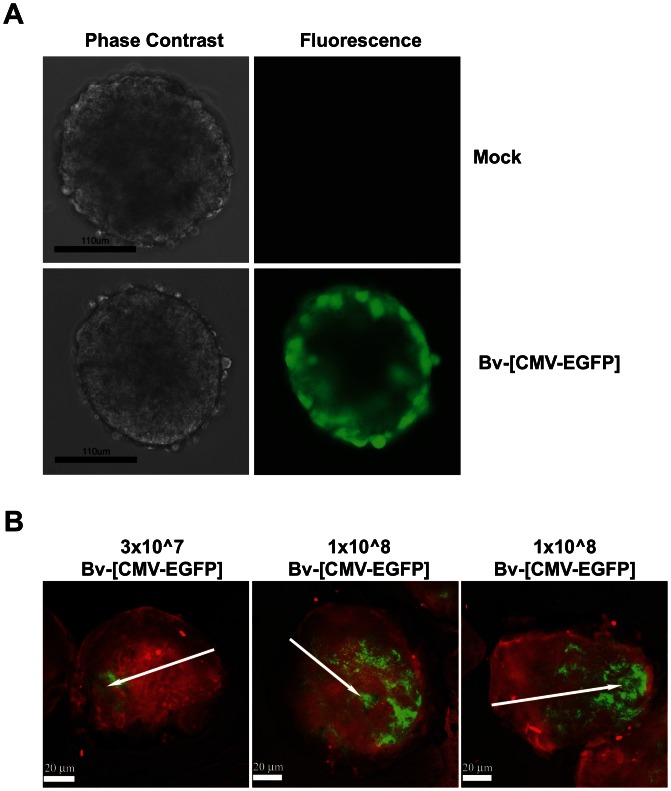
BV transduction of cells growing in three dimensions. (**A**) PC346C spheroids transduced with Bv-[CMV-EGFP] (or mock transduced) at 3 days post-infection. (**B**) PC346C tumours harvested from xenografted mice at 3 days post-infection with BV at 3×10^7^ (one representative image shown) or 1×10^8^ (two representative images shown) pfu per inoculation. Red fluorescence shows cell nuclei (Hoescht), while BV-driven EGFP expression is shown in green. Arrows indicate the site of intratumoural BV injection.

It has been suggested that an effective gene therapy vector must be capable of penetrating through several cell layers in order to achieve a clinically relevant response in the treatment of solid tumours. To characterise the ability of BV to migrate through cell layers, we employed both *in vitro* and *in vivo* three-dimensional cultures. For *in vitro* modelling, hollow spheroids of malignant PC346C prostate cells, which comprised a ‘shell’ of 2–3 cell layers and spontaneously detached from monolayers during routine culture, were incubated with BV-[CMV-EGFP]. At 72 h post-incubation, a highly efficient pattern of cell transduction was observed, with penetration of BV through all cell layers (Figure 3A).

For i*n vivo* modelling, murine prostate cancer xenograft models, generated through orthotopic injection of the PC346C cell line into the dorsolateral prostate of athymic nude mice, were injected intratumourally with BV-[CMV-EGFP] at two different concentrations (3×10^7^ or 1×10^8^ pfu). At 72 h, tumours were resected and analysed by fluorescence microscopy. BV was observed to successfully penetrate into the tumour, and migrate away from the initial site of injection. This was particularly evident following administration of the higher dose of virus (Figure 3B). Thus, even in three-dimensional cultures, BV was capable of achieving a high level of cell transduction.

### Malignant Prostate Cells can be Efficiently Killed Using BV Vectors in a Nitroreductase-based GDEPT Approach

**Figure 4:**
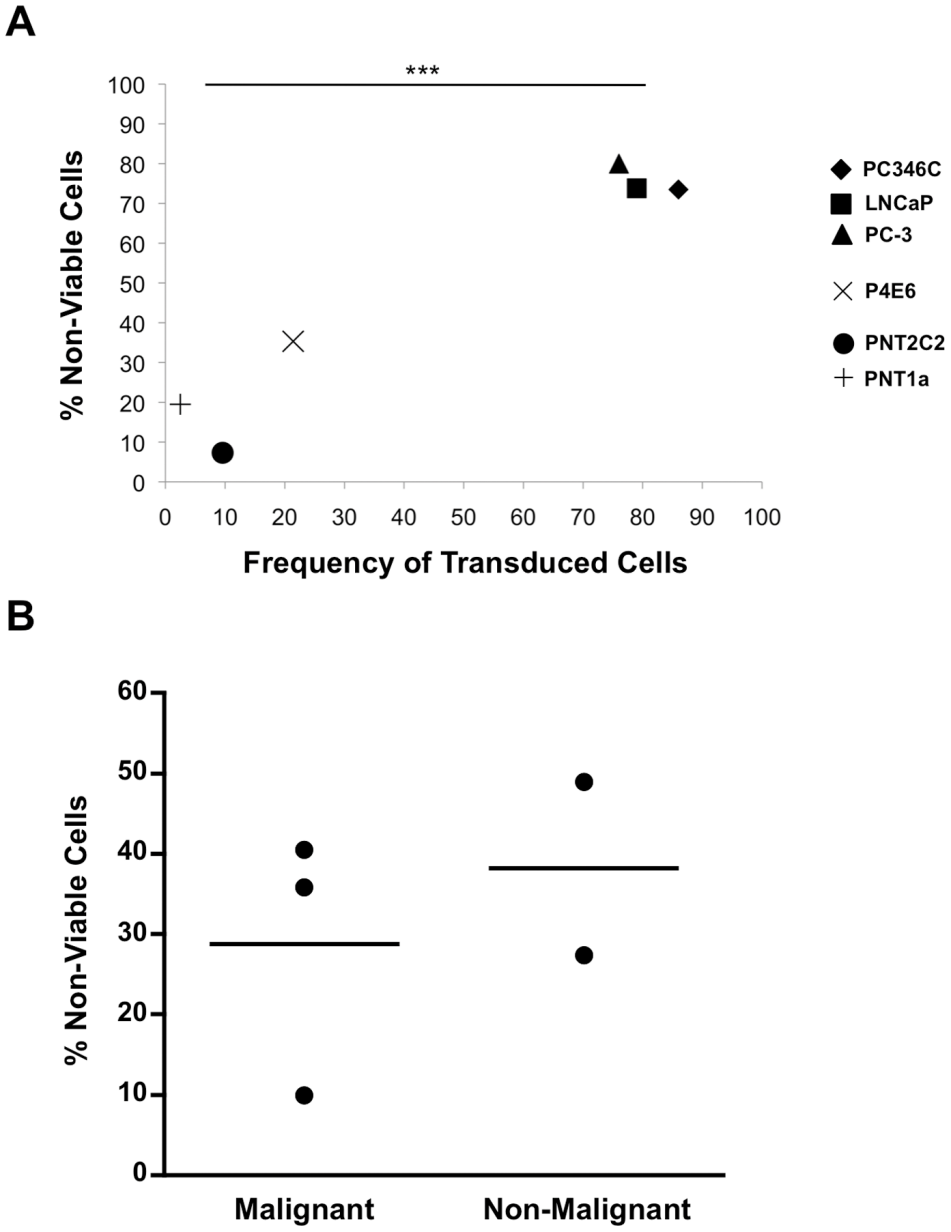
Reduction in cell viability following transduction with a BV expressing a nitroreductase-IRES-EGFP expression cassette and treatment with the pro-drug CB1954. (**A**) Percentage of non-viable cells at 48 h post-transduction with BV-[CMV-NTR-EGFP] (MOI = 500) and treatment with CB1954 in a panel of prostate cell lines, shown relative to frequency of transduced cells and adjusted for untreated controls. Malignant cell lines: PC346C (♦), PC3 (▴),LNCaP (▪), P4E6 (**×**); Non-malignant cell lines: PNT1A (**+**), PNT2C2 (•). Statistics represent t-test between PC346C/PC-3/LNCaP vs. PNT1A/PNT2C2. (**B**) Percentage of non-viable cells at 30 h post-transduction with BV-[CMV-NTR-EGFP] and treatment with CB1954 in individual primary prostate epithelial cell samples (n = 3 malignant and n = 2 non-malignant), adjusted for untreated controls.

In order to test the ability of BV to achieve a cell-lethal outcome, we employed a gene-directed enzyme pro-drug therapy (GDEPT) approach focussed on bacterial *nitroreductase* (NTR), which converts the pro-drug CB1954 (5-(aziridin-1-yl)-2,4-dinitrobenzamide) into a potent DNA cross-linking agent [41]. Therefore, we constructed a BV bearing an expression cassette where the strong mammalian CMV promoter drove expression of mNTR-IRES-EGFP (BV-[CMV-NTR-EGFP]). A panel of malignant and non-malignant prostate cell lines were transduced with BV-[CMV-NTR-EGFP], and cell viability was evaluated following administration of the pro-drug, CB1954. The proportion of non-viable cells, as measured by MTS assay after 48 h, was significantly higher in malignant samples with high metastatic potential (PC346C, PC3 and LNCaP) compared to non-malignant samples (PNT1A and PNT2C2) (P<0.001) (Figure 4A). Early-stage malignant samples (P4E6) fell into the intermediate range of viability. Furthermore, cell viability following pro-drug administration was directly related to the rate of BV transduction (Figure 4A). This analysis was extended into primary patient-derived epithelial cell cultures. Three malignant and two non-malignant samples were transduced with BV-[CMV-NTR-EGFP] and treated with CB1954. At 30 h post-pro-drug administration, cell death in these cultures ranged from 10% to 50%, irrespective of disease status (Figure 4B).

## Discussion

Multiple models of disease have demonstrated that baculovirus has great potential as a novel vector for gene therapy. Here, we have explored its utility for the treatment of human prostate cancer. BV was capable of efficiently transducing a range of prostate cells derived from multiple sources, including *in vitro* and *in vivo* models. BV demonstrated differential transduction of malignant compared to non-malignant prostate cells, an observation that appeared independent of growth rate, since the cell lines in question exhibited similar proliferative capacities (doubling times: PNT2C2 (39 h), LNCaP (34 h) PC-3 (30–34 h) and PNT1a (30 h) [26], [42], [43]). Although a primary mammalian cellular receptor for BV attachment remains unidentified, we speculate that malignancy-associated upregulation of heparan sulphate-modified proteoglycans (HSPGs) on the tumour cell surface [44], [45], which are known to be involved in BV binding [16], [46], plays a role in this outcome. The kinetics of transduction following incubation of prostate cells with BV for increasing lengths of time revealed a difference in the rate of transgene expression between highly susceptible malignant and poorly susceptible non-malignant cell lines, suggesting that differences existed in the process of cellular uptake and nuclear trafficking. However, in the non-malignant cell line PNT1a, we observed viral capsids within the cytoplasm and no obvious defect in the efficiency of nuclear penetration. Further investigation into DNA release from the virus capsid and transcriptional activation is required, since our related research has shown that PNT1A and PNT2C2 cells are also difficult to transfect with non-viral methods (unpublished data).

The primary patient-derived prostate epithelial cell cultures used in this study represented a clinically relevant model for assessing the BV vector [47]. These cultures, like the prostate tumour itself, contained a heterogeneous mix of malignant and non-malignant cells, represented in varying proportions between patient samples. The range of Gleason score tumours we employed demonstrated that BV was able to transduce multiple stages of prostatic disease, yet Gleason score does not determine differentiation status. BV demonstrated a preferential homing ability for basal-like proliferating cells, rather than more differentiated luminal-like cells. This difference in transduction was not unexpected, since many vectors, including retrovirus and adeno-associated virus, are less efficient at penetrating terminally-differentiated cells [48], [49], and even vectors capable of transducing quiescent cells perform better in proliferating cells [50]. Yet, as tissue and tumour growth are driven by an undifferentiated basal cell component, the enhanced ability of BV to target this cell population is likely to be an advantage in achieving longer-term tumour control. Our observation that a substantial proportion of differentiated primary cells could be effectively transduced by BV within four hours is also encouraging. BV is very stable in liquid solution compared to many other viruses, which deteriorate following prolonged incubation at room temperature or higher. Therefore, the need for BV to persist for up to four hours in a tumour environment is highly achievable. We also observed highly efficient BV transduction of prostate stromal cells, a finding that has positive implications for the reduction of prostate tumour burden, since stromal cells encourage tumour growth along the invasion front [40]. Compared to epithelial cells, BV was able to achieve a much higher rate of cellular transduction in a shorter time in stromal cells, which may be related to increased levels of HSPGs in stromal relative to epithelial compartments [51], [52].

The ability of gene therapy vectors to penetrate through cell layers is an important consideration in many clinical situations involving solid tumours. Some vectors, such as the commonly used type C adenovirus, are unable to penetrate the luminal cell barrier, and only rarely diffuse more than one cell layer from an injection needle track unless they are replication-competent [20]. The spread of BV infection within a natural insect host emphasises its ability to penetrate through different cell types [53], [54]. We demonstrated that BV was able to migrate through the tight outer layer of *in vitro* malignant prostate spheroids into second and third cell layers. As these spheroids were hollow, it was not possible to more fully delineate the capacity for complete core penetration. Perhaps most importantly, BV showed a good level of penetration through xenograft tumours, even with a relatively low inoculum of virus particles. With the recent establishment of techniques to produce a highly concentrated clinical-grade BV preparation suitable for human trials [55], an even more thorough coverage of the tumour may be achievable, based on the difference between the two BV doses we evaluated.

While we observed high BV transduction rates in both malignant prostate epithelial and stromal cultures, the use of the NTR/CB1954 GDEPT approach, which exhibits a strong bystander effect, obviates the need for complete transduction coverage [56]. A pronounced bystander effect has previously been observed using this GDEPT approach after transduction of as few as 10% of cells within a culture [57], [58], and has been observed to be even more effective in three-dimensional cultures [59] and therefore tissues, where the permeable active metabolite is transferred via intercellular junctions. Although we analysed the outcomes of pro-drug administration relatively early (48 h for immortalised cell lines or 30 h for non-immortalised primary samples) to maintain cell viability in control wells, we nevertheless observed that the BV-delivered NTR/CB1954 pro-drug system efficiently killed a range of prostate cell types in direct proportion to the frequency of positively transduced cells. In cell lines, there was a differential in the extent of killing between malignant and non-malignant samples, which was less evident in primary samples. This could reflect a practical limitation of our *in vitro* primary cell MTS assay conditions, where the earlier analysis (30 h) of killing necessary to ensure high cell viability in control wells may have attenuated the ability of the BV/NTR/CB1954 GDEPT to achieve maximum effectiveness.

Although this research provides strong evidence demonstrating the feasibility of BV as a gene therapy vector for prostate cancer, it also highlights further areas that must continue to be explored. While administration of BV intratumourally in xenograft models was successful, further studies of the ability of BV to migrate to and infect distal metastases are still required. The extension of the BV-driven GDEPT approach to *in vivo* models of prostate cancer is of great clinical significance, and is currently being explored and developed. Further, the application of the BV-[CMV-NTR-EGFP] transduction/pro-drug conversion system as a low toxicity means to presensitise prostate tumours to the effects of radiation, an approach referred to by Hanna *et al* [60], could be a complementary clinical approach. Nonetheless, the findings presented here support the use of BV as a rational gene therapy vector for human prostate cancer. BV does not suffer from the common restrictions of other viral gene therapy vectors, such as limited DNA capacity and pre-existing virus-specific immune responses, and can easily be modified to incorporate other desirable features, such as targeting and immune shielding. BV displays multiple attributes that recommend its application in a gene therapeutic approach for prostate cancer, and with appropriate regulatory permission, BV has great potential for achieving clinical success.

## Supporting Information

Figure S1 **Induction of differentiation through changes in growth conditions in primary prostate epithelial cell cultures.** Malignant epithelial cells derived from a primary prostate biopsy of Gleason score 8/9 were cultured in either KSFMsup or DH10 medium. (**A**) Morphological differences between cells cultured in KSFMsup or DH10 medium, analysed by phase contrast microscopy. (**B**) Dual immunocytochemistry performed on an alternate batch of the same cells cultured in KSFMsup or DH10 medium. Basal anti-cytokeratins 1, 5, 10 and 14 were conjugated to Alexa 568 (red fluorescence), while luminal anti-cytokeratin 18 was conjugated to FITC (green fluorescence). Cells were counterstained with DAPI to enable nuclear visualisation (blue fluorescence). Co-localisation of basal and luminal fluorescence is shown in yellow (arrow). All images taken at x20 magnification.
(TIF)
