# Supplementary figures and images for: Evaluating Baculovirus as a Vector for Human Prostate Cancer Gene Therapy
Source: PLoS One. 2013 Jun 6;8(6):e65557. doi: 10.1371/journal.pone.0065557 (PMC3675042; doi:10.1371/journal.pone.0065557)

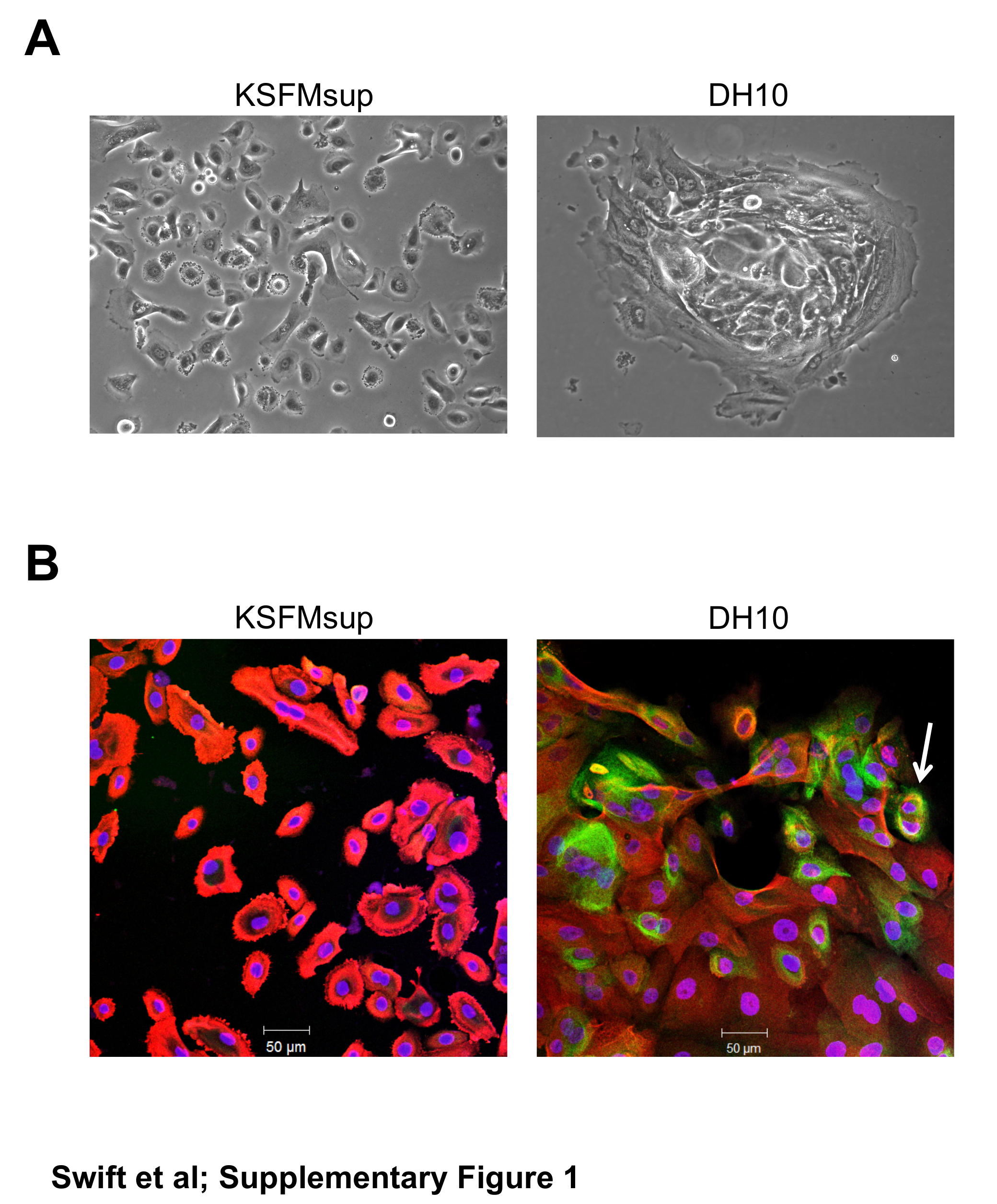

Supplement: Figure S1 — Induction of differentiation through changes in growth conditions in primary prostate epithelial cell cultures. Malignant epithelial cells derived from a primary prostate biopsy of Gleason score 8/9 were cultured in either KSFMsup or DH10 medium. (A) Morphological differences between cells cultured in KSFMsup or DH10 medium, analysed by phase contrast microscopy. (B) Dual immunocytochemistry performed on an alternate batch of the same cells cultured in KSFMsup or DH10 medium. Basal anti-cytokeratins 1, 5, 10 and 14 were conjugated to Alexa 568 (red fluorescence), while luminal anti-cytokeratin 18 was conjugated to FITC (green fluorescence). Cells were counterstained with DAPI to enable nuclear visualisation (blue fluorescence). Co-localisation of basal and luminal fluorescence is shown in yellow (arrow). All images taken at x20 magnification. (TIF) [file pone.0065557.s001.tif]
